# Sweetness Ratings of U.S. Infant Formulas

**DOI:** 10.3390/nu17162602

**Published:** 2025-08-11

**Authors:** Chelsea Olson, Rajesh Kumar, Martin J. Talavera, Christopher E. Anderson, Jennifer A. Hanson

**Affiliations:** 1 Department of Food, Nutrition, Dietetics and Health, Kansas State University, Manhattan, KS 66506, USA; chelseaolson@ksu.edu; 2 Sensory and Consumer Research Center, Kansas State University, Olathe Campus, Olathe, KS 66061, USA; krajesh@ksu.edu (R.K.); talavera@ksu.edu (M.J.T.); 3 Public Health Foundation Enterprise (PHFE) WIC, City of Industry, CA 91746, USA; christophera@phfewic.org; 4 School of Biological Sciences, Louisiana Tech University, Ruston, LA 71272, USA

**Keywords:** infant formula, dietary sugars, taste perception, sensory panel, sweetness

## Abstract

**Background/Objectives**: The U.S. Food and Drug Administration sets nutritional standards for infant formulas. Yet, the carbohydrate components of these formulas can vary markedly in type as well as sweetness intensity. Despite evidence suggesting sweetness can influence intake, limited research exists on the sweetness properties of infant formulas. This study evaluated the sweetness of six commonly used infant formulas in the United States. **Methods**: A sensory panel (*n* = 15) was formed by selecting individuals who achieved a 100% accuracy on three distinct sensory acuity screening tests to assess their ability to detect, differentiate, and quantify various sucrose concentrations. Following training, the panel evaluated each formula during three separate sessions using a sweetness scale from zero to fifteen, where zero represented no sweetness (distilled water) and fifteen represented extreme sweetness (16 g sucrose/100 mL of water). Differences in sweetness were determined using 3-way ANOVA (infant formula, repetition, and panelists) followed by post hoc pairwise comparisons. **Results**: Individual sample sweetness ratings ranged from 0 to 4.5 with a mean rating for all samples of 1.9 (±1.3). Significant differences were detected among the formulas *F*(5, 264) = 96.1, *p* < 0.0001. One formula, a standard milk-based formula, which contains no added non-lactose sugars, was rated significantly sweeter than any of the other formulas while the sweetness ratings for three formulas which all include non-lactose added sugars were significantly lower than that of the other formulas. **Conclusions**: The potential implications of these noted sweetness differences make this an important topic for future sensory, behavior, and health research

## 1. Introduction

Nutrition during early childhood is widely recognized as a matter of profound importance [1,2]. In addition to the more obvious and proximal concerns associated with nutrition in early childhood (e.g., iron deficiency anemia), dietary patterns and food-related behaviors during this life-stage have been shown to track over time [3,4] and influence the incidence of health outcomes throughout the lifespan [5,6,7]. Yet, despite its importance, the intake of food and beverages among infants and toddlers in the United States fails to meet many of the recommendations set forth in the Dietary Guidelines for Americans [8].

Children’s eating behaviors are complex and shaped by a number of factors, including familiarization with foods, exposure, and environment [9]. While the factors that shape eating behaviors are numerous, chemosensory attributes, particularly taste, play a critical role in food selection [10]. Early flavor experiences have been identified as a factor shown to influence intake during later childhood [10,11,12,13]. These taste experiences begin in utero as taste has been shown to be transmitted to the fetus through amniotic fluid [12].

Infants and children exhibit a heightened innate preference for sweetness compared to adults [14]. Research examining children’s affinity for sweetness has identified a positive correlation between sweetness preference and body mass index (BMI) [15,16,17]. Similarly, infants have been observed to exhibit a greater intake of sweeter liquids, though studies in this area remain limited. For example, Maller et al. [18] found that neonates (n = 192) consumed larger volumes of fructose and sucrose water solutions compared to lactose and glucose solutions, and this observed intake pattern aligns with the higher sweetness levels of fructose and sucrose relative to lactose and glucose. The relationship between increased intake of sweet foods and hedonic hunger offers a compelling explanation of such behavior. Highly palatable foods, including those rich in sweetness, can stimulate the dopamine reward circuitry of the brain [19]. This activation of the reward system can override homeostatic satiety mechanisms, driving motivational feeding behaviors and leading to increased caloric consumption [20]. Research suggests that even infants can be influenced by hedonic hunger, highlighting its potential role in early feeding behaviors [21].

The first postnatal exposure an infant has to sweetness occurs through either infant formula or breastmilk, which should serve as the sole source of dietary intake for at least the first four months [22]. This period has been shown to involve increased neural sensitivity to environmental influences, leading to a stronger impact from exposure to sweetness. This underscores the importance of evaluating the flavor profile of infant formulas. Potential flavor variations arise from differences in composition of carbohydrates, proteins, fats, and inclusion of additives, all of which impact sweetness perception. Infant formula sweetness matters, as flavor has been identified as impacting food related behaviors in infancy and into childhood [10,11,12,23,24].

Acknowledging the limitations of infant formula in replicating the health and nutritional properties of breast milk, the American Academy of Pediatrics and the World Health Organization (WHO) recommend exclusive breastfeeding for the first six months of life [25,26]. However, nearly 20% of infants in the U.S. are given formula by two days of age, and only 24.9% are fed in accordance with the six-month breastfeeding guidelines [27]. The U.S. Food and Drug Administration (FDA) regulates infant formula to assure nutritional and safety standards are meet [28]. Yet, the components of infant formulas can vary markedly, and the carbohydrate content which may contain added sugars remains largely unregulated. In fact, many infant formulas in the U.S. contain added non-lactose sugars [29]. Given the widespread reliance on formula, the composition of infant formula, particularly its sugar configuration and sweetness value, remains a critical public health concern.

Added sugars in infant formula are a source of carbohydrate and contribute to the formula sweetness perception and palatability. Many of these added sugars, which are either entirely absent or present in different quantities compared to breastmilk, are included to provide an energy source for growing infants [30,31,32]. The carbohydrate components of infant formula are known to vary in sweetness intensity, and recent research has identified a link between infant formula carbohydrate composition and anthropometric measures during infancy and early childhood [33,34,35].

Despite evidence suggesting that sweetness can influence intake, limited research exists on the sweetness properties of infant formulas and their potential impacts on infant and child health. Additionally, while a small number of studies have evaluated various sensory attributes of infant formula [36,37], to our knowledge, no studies have specifically quantified the sweetness level of U.S. infant formula.

The objective of this study was to evaluate the sweetness ratings of six of the most commonly used infant formulas in the United States from the two manufacturers that hold all Special Supplemental Nutrition Program for Women, Infants, and Children [WIC] state-level infant formula contracts. Establishing the sweetness levels of infant formulas is an essential initial step toward understanding the potential relationship between sweetness and subsequent dietary and health outcomes in infants. This is particularly true for newer products such as the lactose-reduced options. Additionally, this study aimed to provide valuable insights into the sensory attributes that contribute to flavor perception in infant formulas. These findings may help guide healthcare professionals and consumers in making more informed decisions when selecting infant formulas.

## 2. Materials and Methods

### 2.1. Sensory Lab Protocols

The sensory panelists’ screening, training, and evaluation activities were conducted at the Sensory and Consumer Research Center at the Kansas State University-Olathe Campus (K-State Olathe). Stringent environmental controls were implemented during all sensory activities. These measures ensured a quiet environment free from distractions or interruptions, comfortable surroundings, controlled lighting, and regulated air conditioning to maintain optimal temperature, humidity, and air purity. Additional precautions included maintaining neutral facial expressions, thorough rinsing procedures, and prohibiting the use of phones during testing sessions. Panelist screening, training and sample tasting occurred over three days.

### 2.2. Sensory Panel Recruitment

To form the sensory panel, 200 individuals were contacted through a large database maintained by the center. The respondents were screened using a questionnaire that included questions on sex, age, food allergies, smoking, and willingness to taste infant food and formula. Also, participants should not have taken part in any consumer research and/or sensory taste test in the past three months anywhere in the United States. Eighteen individuals met the initial screening criteria and were selected for panel training and descriptive analysis. At the outset of the study, the research team provided detailed instructions and guidelines. Personal hygiene protocols included abstaining from wearing lipstick, perfumes/colognes, or fragrant hair sprays, as well as refraining from smoking, eating, and drinking for at least one hour prior to participation. In addition, participants provided written informed consent and were compensated for their time. Payment was received upon the completion of the sensory evaluation on the third day. This study was conducted under the Kansas State University Institutional Review Board (IRB) approval #05930 (25 July 2011) using approved protocols. All procedures were performed in compliance with relevant laws and institutional guidelines.

### 2.3. Infant Formula Selection

Based on prevalent WIC contracts, which account for the majority of infant formula purchased in the United States, six individual formulas sold under two brands were selected for testing. The selected formulas included two standard milk-based formulas, two reduced-lactose formulas with added corn syrup solids, a lactose-reduced formula with added rice, and a lactose-free soy-based formula. See Table 1 for additional details regarding the formulas selected for analysis.

### 2.4. Panel Formation

During the morning of day one, each of the 18 individuals who met the initial screening criteria underwent a series of three distinct sensory acuity screening tests to assess their ability to detect, differentiate, and quantify various sucrose concentrations (i.e., sweetness levels) in aqueous solutions (distilled water).

1. Sweetness Intensity Ranking Test: Participants were presented with four different sucrose solutions, ranging from 0 g sucrose/100 mL of water to 5 g sucrose/100 mL of water. They were asked to rank the solutions in order of perceived sweetness.
2. Triangle Test: Participants were given three solutions; two sucrose (2 g sucrose/100 mL of water) samples and one water solution (0 g sucrose/100 mL). They were tasked with identifying the sample that was different from the other two.
3. Tetrad Test: Participants received four sucrose samples, two of one concentration (3 g sucrose/100 mL of water) and two (5 g sucrose/100 mL of water) of another concentration. They were required to identify and group samples which were the same.

Only individuals who achieved a 100% accuracy across all three screening tests were selected to continue as panel members for the sensory analysis. This selection process resulted in the elimination of three participants, leaving a final panel of 15 members. The panel members were predominantly Caucasian (13 members), with one Asian American and one African American. The sex distribution consisted of five males and ten females. The age of the panel members ranged from 39 to 68 years, with an average age of 56.1 years. None of the panel members were smokers, and no known food allergies were reported.

### 2.5. Panel Descriptive Training

Following selection, panel members participated in descriptive training to assess sweetness levels in the afternoon of day one. Three reference solutions were prepared with different sugar concentrations: 0 g sucrose/100 mL of water, 2 g sucrose/100 mL of water, and 4 g sucrose/100 mL of water. These concentrations were determined by the sensory lab after conducting a technical tasting session of infant formula samples by five highly trained sensory scientists. All infant formula samples were tasted along with five reference sucrose solutions ranging from sweet = 0.0 (0 g sucrose/100 mL of water) to sweet = 5.0 (5 g sucrose/100 mL of water) to represent an appropriate range of sweetness levels for the formulas.

For panel orientation and training only three infant formula samples that represented wide sweetness intensity range were prepared from powder according to label directions. Each sample was individually compared to the reference sucrose solutions. During the comparisons, panel members engaged in discussions and received training focused on assessing only the sweetness intensity in each formula.

Panel members practiced using a descriptive training ballot designed to measure sweetness intensity. The paper ballot featured a sweetness ranking scale from 0 to 15, where 0 represents no sweetness (distilled water) and 15 represents extreme sweetness (16% sucrose solution or 16 g sucrose/100 mL of water). The scale was marked in 0.5 increments. This 0 to 15 scale described by DuBois et al. [38] provides a simple and unambiguous method for communicating sweetness results [39] and is considered a universal scale. Training was provided to panel members on how to accurately evaluate and rank the sweetness of each sample using this scale.

### 2.6. Sensory Evaluation

On day two, panel members participated in two sensory evaluation sessions, held at 10:30 AM and 2:30 PM CST. To quantify sweetness, panel members used the 0 to 15 scale they were trained on the day prior to rate formula sweetness relative to standard reference solutions of sucrose. Seven formula samples were prepared for each of these sessions. The samples consisted of one sample of each of the six study formulas plus one repeat sample. All samples were prepared within ten minutes of testing. The powdered formulas were mixed in glass bottles (650 mL SodaStream Glass Carafe) using room temperature distilled water and shaken according to the label directions. Each formula sample was assigned a three-digit randomized code (e.g., 357), the three-digit sample codes changed for each session to prevent familiarity with repeated formulas. The prepared formulas were transferred into 118 mL disposable polystyrene translucent plastic souffle cups (Dart, Mason, MI, USA), covered with clear lids, and labeled with the appropriate three-digit code. Panel members had five minutes to taste and rate the sweetness of each formula and provide any additional comments. They were given an additional two minutes between samples to cleanse their palates and remove any lingering tastes. The samples were evaluated following a sequential monadic serving design and were presented based on a complete randomized balance design.

Nabisco Premium unsalted crackers and plain water were used as palate cleansers. Table settings included a spitting cup, a pencil, and a descriptive paper ballot. Panel members had access to three reference solutions for sweetness: 0 g sucrose/100 mL of water, 2 g sucrose/100 mL of water, and 4 g sucrose/100 mL of water. The descriptive ballot included sections for the sample number, a sweetness scale ranging from 0 to 15 in 0.5 increments, and a comment section where participants could add additional remarks about taste, flavor, texture, mouthfeel, and other sensory attributes. On day three, panel members returned to the center for the last sensory analysis session. All of the above protocols were followed. In all, each participant evaluated all six infant formulas with three replications in three different sessions over two days.

In descriptive sensory science, a trained panel of 15 assessors, each performing three assessments per product, meets established best-practice standards, delivering robust and valid statistical results by facilitating ANOVA, assessing within- and between-assessor consistency, and ensuring reliable discrimination across products [40,41,42]. Furthermore, recent research suggests that two and even one assessment may be sufficient to provide valid information in most cases, depending on the level of training of the panel [43].

### 2.7. Analysis

A three-way analysis of variance (ANOVA) using Tukey’s Honestly Significant Difference was performed to identify significant differences (*p* < 0.05) among the samples based on sweetness intensity ratings. Panelist and replications were the other main effects. Statistical analysis was performed using XLStat software version 2023.1.4 (Lumivero, Denver, CO, USA). Sensory panel member comments were compiled and organized by formula type, panel member number, and replication.

## 3. Results

**Figure 1:**
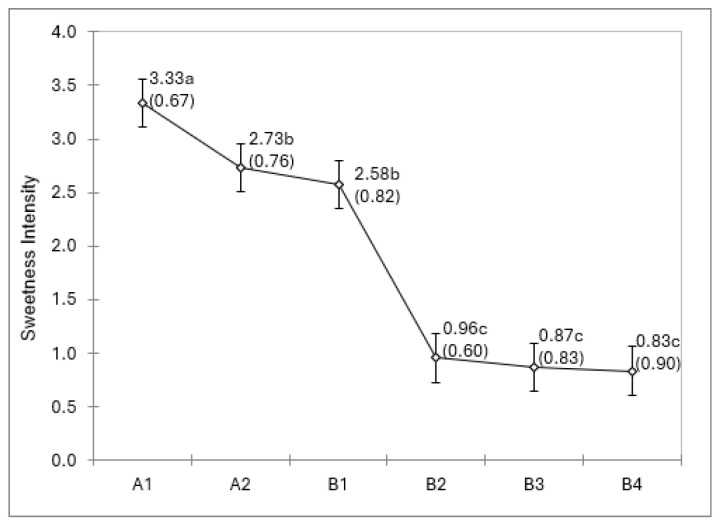
Infant formula mean sweetness ratings and standard deviations. a–c formulas identified with different letters differ significantly (*p* < 0.05).

The sensory panel analysis revealed a range of sweetness levels with individual sample ratings ranging from 0 to 4.5. The mean sweetness rating for all samples was 1.9 (±1.3). The results of the ANOVA for sweetness ratings revealed significant differences among the infant formulas *F*(5, 264) = 96.1, *p* < 0.0001. Post hoc pairwise comparisons found the sweetness rating for A-1 significantly higher than that of the other infant formulas and sweetness ratings for B-2, B-3, and B-4 significantly lower than that of the other formulas (Figure 1).

Sensory panel member comments indicated a range of sensory attributes were detected and noted. Common phrases used to describe the infant formulas included “grassy”, “creamy”, “frothy” or “foamy” and “aftertaste”. A full listing of comments can be found in Table 2.

## 4. Discussion

The objective of this study was to evaluate the sweetness levels of six commercially available infant formulas. Using a trained sensory panel, three independent evaluations were conducted over two consecutive days. The results revealed significant differences in formula sweetness ratings. In addition, panelist feedback indicated a range of sensory attributes were perceived. This study is the first, to our knowledge, to employ a trained sensory panel to systematically assess and rank the sweetness levels of infant formulas.

The infant formulas with the lowest sweetness ratings (i.e., B-2, B-3, and B-4) were all formulas that contain non-lactose added sugars. This finding was unexpected as lactose, with a relative sweetness ranging from 15 to 40, is the least sweet among the common monosaccharides and disaccharides. In comparison, other carbohydrates such as glucose (50–75) and sucrose (100) exhibit higher sweetness levels [44]. Among these, sucrose has the highest relative sweetness value of the added sugars commonly found in the infant formulas analyzed in this study. As a disaccharide, sucrose consists of glucose and fructose linked by a glycosidic bond [45].

Glucose polymers are frequently added to infant formulas, particularly in lactose-reduced or lactose-free varieties. These polymers, which include corn syrup solids and maltodextrin, contain multiple glucose molecules linked together [45,46]. Liquid corn syrup is produced through the hydrolysis of corn starch and contains shorter chains of glucose, maltose, and free glucose molecules [45]. In turn, corn syrup solids are produced by dehydrating liquid corn syrup [47]. Corn syrup solids vary in composition and have been reported to exhibit 35–60% of the sweetness of sucrose [47].

Maltodextrins are produced through enzymatic or acid hydrolysis of starch and share a molecular structure similar to other glucose polymers. However, the source of the starch, which is commonly corn, potato, wheat, or rice, is typically not disclosed on product labels [46]. While the sweetness levels of maltodextrins vary, they are generally regarded as low to moderately sweet [45,46].

In addition to maltodextrins, rice starch was included in the anti-reflux formula. Rice starch undergoes extensive processing, involving the milling of rice kernels, soaking in sodium hydroxide, grinding into flour, and dehydration [48]. Rice starch is used to thicken infant formulas [49]. Because it is considered bland or flavorless [50], it likely contributes minimally to the sweetness of infant formula. Sensory evaluations of the anti-reflux formula revealed textural descriptors such as “foamy,” “frothy,” “thick,” and “viscous.” Texture can influence flavor perception, with more viscous products often exhibiting lower perceived sweetness [51,52]. Thus, the textural modifications introduced by rice starch and other ingredients may have also influenced the panelists’ sweetness perceptions.

Non-digestible carbohydrates (i.e., prebiotics) were included in four of the six infant formulas analyzed in this study, specifically polydextrose, galactooligosaccharides, and/or fructooligosaccharides. Polydextrose is a non-digestible, randomly linked polymer of glucose characterized by a lack of sweetness [53]. Its relative sweetness is approximately 5% that of sucrose [54]. Galactooligosaccharides, described as having mild sweetness ranging from 30% to 60% of that of sucrose, are a group of galactose-containing oligosaccharides with chemical structures that vary by chain length, branching, and glycosyl linkages [55]. In comparison, short-chain fructooligosaccharides are carbohydrates composed of a single glucose molecule (a six-carbon sugar) bonded to two, three, or four additional fructose molecules [56]. These compounds are noted for their mild sweet taste, providing approximately one-third the sweetness of sucrose [57]. While these prebiotics likely contribute only mild sweetness independently, their inclusion may influence the overall sweetness profile of the infant formulas.

Infant formulas comprise a complex combination of fats, proteins, carbohydrates, vitamins, minerals, and additional additives. The unexpected sweetness rating results, as well as the sensory panel comments, suggest the sensory characteristics of individual ingredients, as well as their interactions, likely contribute to the perceived sweetness differences observed. The type of protein used in infant formulas significantly influences the perception of sweetness, particularly in formulas containing partially or fully hydrolyzed proteins. Hydrolysis is used to break cow’s milk proteins into smaller peptides and free amino acids [58,59]. While this breakdown improves protein digestibility [58], it also results in a distinct bitter flavor profile [59]. The intensity of bitterness is linked to the degree of hydrolysis [60] with infant formulas containing extensively hydrolyzed proteins being characterized as more bitter and less palatable compared to less hydrolyzed or non-hydrolyzed formulas [24,60,61]. For example, an analysis of 25 hydrolyzed and amino acid-based formulas by Miraglia Del Giudice et al. [62] identified significant variations in taste, which were attributed to differences in nitrogen sources, peptide composition, lipid content, and lactose levels. To counteract the bitterness associated with hydrolyzed proteins, manufacturers may incorporate glucose polymers and/or sucrose to enhance palatability.

The primary protein source in soy-based infant formulas is soy protein isolate [63], which is produced from dehulled soybeans by removing the majority of non-protein components. However, this process may leave behind some impurities (e.g., phytates and phenolic substances) which impair sensory quality. While detailed dehulling of the beans, thorough extract clarification, and repeated washing of the soy curd reduce the impurities, they are not removed entirely [64]. During sensory evaluations of the soy-based infant formula (i.e., B-4), panelists reported experiencing “grassy”, “grainy” and “earthy” flavors. These sensory attributes may be linked to several compounds, including isoflavones, which have been described as beany and astringent [65]. Previous sensory research on soy milk have identified qualities such as “pea-like”, “earthy”, and “green, which are primarily attributed to the soy content [66]. Interestingly, in an older study several soy-based formulas were perceived as sweeter than milk-based formulas, a finding that contrasts with the results of this study [67]. However, it should be noted that this older study was conducted using Canadian infant formulas and published over 45 years ago.

The milk-based infant formulas in this study included milk protein isolate, nonfat milk, and/or whey protein concentrates. Milk protein isolates are extensively processed to remove most of the non-protein particles (e.g., lactose, minerals). Whey protein concentrates are produced through the ultrafiltration of whey, a process that retains a high concentration of protein [68]. Milk proteins are not flavorless and exhibit a wide array of flavors [69]. Whey protein concentrates have been described as having sweet aromatic notes, as well as flavors such as “cardboard/wet paper”, “pasta water”, and “cooked/milky” [70]. Some whey concentrates have exhibited the basic taste of bitterness and the feeling factor of astringency [70]. The specific type of protein incorporated into infant formulas might play a critical role in influencing the perceived sweetness and overall sensory characteristics of the formula.

Lipids represent another variable influencing the taste perception of infant formulas. Common fats used in infant formulas include vegetable oils, fish oils, algae oils, fractionated lipids, and egg phospholipids [71]. The infant formulas analyzed in this study incorporated varying combinations of high oleic sunflower oil, high oleic safflower oil, soy oil, and coconut oil, with four formulas also containing palm olein (the liquid fraction of palm oil). Sunflower oil, safflower oil, soy oil, and refined palm oil are generally regarded as neutral or mild in flavor [72,73,74]. Coconut oil’s sensory attributes depend on the degree of processing and can range from “a salty taste with no perceivable flavor” to a “detectable sweet taste and nutty flavor” [75]. Although these fats generally contribute mild taste characteristics, it remains unclear how their specific combinations may influence the perceived sweetness of the formulas.

Lipids in infant formula are susceptible to oxidation during processing or storage, posing a potential challenge to product quality. Lipids containing higher levels of polyunsaturated fatty acids (PUFAs) are especially susceptible to peroxidation, a process that generates unpleasant and potentially toxic by-products [76]. Rancidification occurs through oxidation or hydrolysis when fats are exposed to light, air, or heat, resulting in the formation of aldehydes, ketones, and other secondary products which contribute distinct undesirable flavors and aromas [77]. Infant formulas are vulnerable to rancidity due to their lipid composition. Among the formulas analyzed in this study, fat accounted for roughly 45–50% of each formula’s total energy with a notable portion coming from polyunsaturated fatty acids. The extent of oxidation is commonly assessed by peroxide values, which indicate the presence of oxidized fatty acids and serve as a measure of rancidity in food products [78]. The susceptibility to lipid degradation in infant formulas is influenced by the specific combination of metal ions (e.g., copper and iron), PUFAs, and vitamins present. Such degradation may alter the flavor profile of the formula, potentially interfering with the perceived sweetness.

All six formulas evaluated by the sensory panel contained a combination of docosahexaenoic acid (DHA) and arachidonic acid (ARA) sources. The average DHA content in U.S. infant formulas is 12.6 mg per 100 kcal [79]. In the formulas examined in this study, DHA was sourced from *Schizochytrium* sp. oil and *Crypthecodinium cohnii* oil, while ARA was derived from *Mortierella alpina* oil. These oils are generally considered neutral in flavor. However, both are susceptible to oxidation, which can lead to the development of “off” odors and flavors such as strong marine or fishy odors and flavors [80,81]. Without specific data on the amounts, ratios, and oxidation levels, it is difficult to draw definitive conclusions regarding their impact on the flavor of the infant formulas. Nevertheless, the inclusion of these fatty acids is an important factor to consider in understanding the overall sensory profile of the formulas.

There are several limitations to this study. First, the study was unable to obtain a representative sample of the target population. Assessing infants’ perception of sweetness or liking is challenging, and some studies have relied on indirect measures such as intake, ingestion ratio, duration of eating, facial expressions, and subjective ratings from mothers [82,83,84,85,86]. We used an adult panel with an average age of 56.1 years to assess the sweetness level. Adults and infants have different taste perceptions due to developmental differences in taste buds and sensory systems. Infants have a higher concentration of taste buds and are thought to be more sensitive to certain flavors, including sweetness [87,88].

Additionally, researchers have used different methods to assess sweetness perception in adults and infants, which complicates the comparison of adult sweetness perceptions to infant preferences. Researchers studying infants rely on nonverbal cues, such as facial expressions (e.g., smiling, grimacing) and physiological responses like sucking rate, to assess sweetness, whereas adults can provide feedback using verbal descriptors or numerical scales. Bias is another concern with any descriptive panel; adults have more experience and preconceived notions about sweetness, which can influence their judgments. They might subconsciously compare the sweetness of infant formula to other sweet foods they are familiar with, leading to biased assessments. Furthermore, some adults are more sensitive to sweetness than others [89], and several genetic variations related to perceptions of sweetness and bitterness have been identified [90].

## 5. Conclusions

Our findings identified important differences in sweetness among six of the most commonly used infant formulas in the United States. Somewhat surprisingly, the infant formulas with the lowest sweetness ratings were all formulas that contain non-lactose added sugars. This finding, in addition to panel member comments, suggests that the sensory characteristics of individual ingredients, as well as their interactions, likely contributed to the sweetness differences. This study contributes to the limited body of research on the sweetness profiles of infant formulas, establishing a baseline understanding of formula sweetness. An evaluation of infant formulas beyond those available in the United States would add a global perspective. In addition, future research should investigate the potential health implications of consuming sweeter formulas, particularly in relation to infant feeding behaviors, such as overeating or developing preferences for sweet foods, which could contribute to an increased risk of childhood obesity.

## Figures and Tables

**Table 1:** Infant formulas and carbohydrate sources.

Brand-Formula	Type	Carbohydrate Source
A-1	Standard, milk-based	Lactose
A-2	Milk-based, lactose-reduced formula	Corn syrup, sucrose
B-1	Standard, milk-based	Lactose
B-2	Added rice to reduce spit-up, lactose reduced	Rice starch, lactose, maltodextrin
B-3	Partially hydrolyzed, milk-based, lactose reduced	Corn syrup, lactose
B-4	Soy protein-based	Corn syrup

**Table 2:** Sensory panel comments regarding the sensory attributes of each infant formula sample.

Panelist	Replication	A-1	A-2	B-1	B-2	B-3	B-4
1	1	pleasant aftertaste	pleasant taste	pleasant taste	bitter taste	mellow taste	slight aftertaste
	2	strong smell	stronger smell, little aftertaste	slightly sweeter		neutral taste	slightly sour
	3	strong smell, very little aftertaste	strong smell	very little aftertaste, slightly strong smell	foamy	neutral taste	strong smell, some aftertaste, unpleasant taste
2	1						flavor seems balanced
	2						
	3					flavor and sweetness seems balanced	grass-like flavor
3	1	thicker	sweetness not really noticeable	aftertaste, thinner consistency	lighter	no sweetener, more like milk	frothy, not sweet
	2		a bit sweet		frothy, bad aftertaste	much lighter	
	3	tasted closest to sweetened milk	smell is not bad		frothy/foamy	sour	tasted like dirt
4	1	creamy	creamy	creamy	frothy	watery, bitter	grassy odor
	2	creamy	creamy	creamy	frothy	very bitter aftertaste	grainy, watery
	3	pleasant	creamy	creamy	frothy, watery	bitter, sour aftertaste	grassy taste
5	1				foamy, difficult to salivate		
	2				foamy		
	3				foamy texture		
6	1		smooth texture, not gritty				
	2						
	3				very thick consistency		horrible aftertaste
7	1	creamy	nice smooth texture	a little watery	foamy		thin
	2	smooth	not tasty though sweeter, creamier	good texture	thick and frothy	frothy	not sweet
	3	milky	fine texture	thinner texture	very foamy	bitter	not tasty
8	1	good consistency	unpleasant odor	liked sweetness and flavor more	thick, frothy	no aftertaste	flavor was off, very thin
	2	good sweetness		perfect sweetness level	frothy	decent taste with no aftertaste	not frothy or too thick, left aftertaste
	3	bad flavor	good sweetness, no overpowering flavors	bad odor, aftertaste	frothy, consistency overpowers taste/sweetness level	mild flavor, needs more sweetness	bland, wheat taste
9	1	thin		pleasant flavor	thick	odd metal taste	earthy taste
	2	thin, creamy taste	pleasant taste	pleasant taste, sweet grass taste	thick, a bit sweet	thin with metal aftertaste, a little bitter	thick, vitamin taste
	3		pleasant taste, thin	pleasant, sweet grassy aftertaste	thick, foamy, not pleasant	bitter and taste of metal	different, not sweet, not unpleasant
10	1		smells bad		sour bitter		powdery
	2		a little bitter		foamy	horrible	
	3	strong aftertaste		bad aftertaste	foamy, bad taste	worst taste, bitter, acidic, spicy	horrible, spicy, bitter
11	1						
	2						
	3						
12	1					mild aftertaste	
	2				foamy, strong smell		
	3				too thick and foamy	sour, no sweetness at all	
13	1				foamy		grassy, pine nutty taste
	2				too foamy	sour	
	3					bad aftertaste	smelled like a plant
14	1			smooth, creamy	foamy	bitter aftertaste which lingers	smooth
	2			creamy mouthfeel, smooth		bitter aftertaste	aftertaste
	3			creamy	foamy	little bitter	
15	1	watery, noticeable odor	watery, smells sour, tastes green	neutral flavor, watery	viscous, slight odor	slight aftertaste, sour and watery	tastes like grass, odor less desirable
	2	slight vegetable taste	vegetable taste, milk like consistency	milk-like consistency, no bitterness	viscous, slight displeasing odor	neutral taste, milk-like consistency	darker, watery
	3	milk-like consistency	slight sour smell, creamy, sour aftertaste	sweet smell, no aftertaste, watery	thick consistency, no bitterness, no aftertaste, slight smell	watery, bitter	vegetable smell, creamy, slight aftertaste

## Data Availability

A copy of all of the data used in this study may be obtained from the corresponding author who has full access to the data reported in this manuscript.

## Abbreviations

The following abbreviations are used in this manuscript:

FDA	The U.S. Food and Drug Administration
WHO	World Health Organization
WIC	Special Supplemental Nutrition Program for Women, Infants, and Children
ANOVA	Analysis of variance
DHA	Docosahexaenoic acid
ARA	Arachidonic acids
